# Editorial: Mining Scientific Papers: NLP-enhanced Bibliometrics

**DOI:** 10.3389/frma.2019.00002

**Published:** 2019-04-30

**Authors:** Iana Atanassova, Marc Bertin, Philipp Mayr

**Affiliations:** ^1^CRIT, Université de Bourgogne Franche-Comté, Besançon, France; ^2^ELICO, Universié Claude Bernard Lyon 1, Lyon, France; ^3^GESIS - Leibniz Institute for the Social Science, Cologne, Germany

**Keywords:** text mining, scientific papers, scientometrics, natural language processing, computational linguistics, citation content analysis, academic search

## 1. Introduction

The Research Topic on “NLP-enhanced Bibliometrics” aims to promote interdisciplinary research in bibliometrics, Natural Language Processing (NLP) and computational linguistics in order to enhance the ways bibliometrics can benefit from large-scale text analytics and sense mining of papers. The objectives of such research are to provide insights into scientific writing and bring new perspectives to the understanding of both the nature of citations and the nature of scientific papers and their internal structures. The possibility to enrich metadata by the full-text processing of papers offers a new field of investigation, where the major problems arise around the organization and structure of text, the extraction of information and its representation at the level of metadata.

Recently, the ever growing availability of datasets and papers in full text and in machine-readable formats has made possible a change in perspective in the field of bibliometrics. From preprint databases to the Open Access and the Open Science movements, the development of online platforms such as ArXiv, CiteSeer or PLoS and so forth, largely contribute to facilitating the experimentation with datasets of articles, making it possible to perform bibliometric studies not only considering the metadata of papers but also their full text content.

The field of NLP offers methodological frameworks and tools for the full text processing of papers that can enlighten bibliometric studies. Some of the open source tools for text processing that have been recently applied to such tasks include NLTK, Mallet, OpenNLP, CoreNLP, Gate, CiteSpace, AllenNLP, and others. Many datasets are now freely available for the community: e.g., PubMed OA, CiteSeerX, JSTOR, ISTEX, Microsoft Academic Graph, ACL anthology, etc. The further developments in this field of study need producing annotated corpora and shared evaluation protocols in order to enable the comparison between different tools and methods. The development of such resources is an important step to making scientific reproducibility possible.

## 2. Papers in This Research Topic

The seven papers published in this Research Topic were all reviewed by two independent reviewers.

In the paper “Is the Abstract a Mere Teaser? Evaluating Generosity of Article Abstracts in the Environmental Sciences,” Ermakova et al. examines the abstracts of scientific papers. In fact, the abstract points out the information that is the most important for the reader and is often used as a proxy for the content of an article. The authors propose the GEM score that measures the representativeness of an abstract or its “generosity.” To obtain this score, sections in the papers were weighted according to their importance to the reader and sentences in the abstracts were assigned to different sections based on their similarity with the content of the sections. More than 36,000 papers in environmental sciences, retrieved from the ISTEX database, were processed to observe the trends in the GEM score over an 80-year period of time. The results show that abstracts tend to be more generous in recent publications and there seems to be no correlation between the GEM score and the citation rate of the papers.

In the paper “The Termolator: Terminology Recognition Based on Chunking, Statistical and Search- Based Scores,” Meyers et al. propose an open-source high-performing terminology extraction system called Termolator which utilizes a combination of knowledge-based and statistical components. The Termolator tool includes chunking that favors chunks containing out-of-vocabulary words, nominalizations, technical adjectives, and other specialized word classes and supports term chunk ranking. The authors analyse the effectiveness of all involved components to the overall system's performance and compare their Termolator system with a terminology extraction system called Termostat. They use a gold standard consisting of manually annotated instances of inline terms (multi-word nominal expressions) of different types of documents (e.g., patent, journal article).

In the paper “Deep Reference Mining From Scholarly Literature in the Arts and Humanities,” Rodrigues Alves et al. work on a deep learning architecture for the detection, extraction and classification of references within the full text of scholarly publications. The authors explore word and character-level word embeddings, different prediction layers (Softmax and Conditional Random Fields) and multi-task over single-task learning components. Their experiments are based on a published dataset of annotated references from a corpus of publications on the historiography of Venice (books and journal articles in Italian, English, French, German, Spanish and Latin) published from the nineteenth century to 2014. In the evaluation the authors show the relative positive contribution of their character-level word embeddings. The authors release two implementations of the architecture, in Keras and TensorFlow, along with all the data to train and test. Their results strongly support the adoption of deep learning methods for the general task of reference mining.

In the paper “Temporal Representations of Citations for Understanding the Changing Roles of Scientific Publications,” He and Chen propose an analysis of the temporal characteristics of citations in order to represent the dynamic role of scientific publications. For this purpose, they study and compare different types of citation contexts in order to identify articles that play important role in the development of science. The proposed methods can have different applications, such as improving citation-based techniques at the individual or collective level, but also improving recommendation systems dedicated to information retrieval by identifying articles of importance or interest.

In the paper “Resolving Citation Links With Neural Networks,” Nomoto presents a novel way to tackle the citation resolution through the application of neural network models and identifying some of the operational factors that influence their behavior. The author introduces the notion *approximately correct targets* which is “an idea that we should treat sentences that occur in the vicinity of true targets as equally correct, whereby we try to identify an area which is likely to include a true target, instead of finding its exact location.” Experiments in the paper are conducted using three datasets developed by the CL-SciSumm Shared Task (ACL repository) and a cross validation style setup.

The two papers “The NLP4NLP Corpus (I and II): 50 Years of Publication, Collaboration and Citation in Speech and Language Processing” by Mariani et al. and Mariani et al., present the results of an extensive study of a dataset in the field of Natural Language Processing (NLP) and Spoken Language Processing (SLP) for the period 1956–2015. The authors investigate various trends that can be observed from the publications in this specific research domain. The study is presented in two companion papers that each provides a different perspective of the analysis. The first paper describes the corpus and presents an overall analysis of the number of papers, authors, gender distributions, co-authorship, collaboration patterns and citation patterns. The second paper investigates the research topics and their evolution over time, the key innovative topics and the authors that introduced them, and also the reuse of papers and plagiarism. Together, the two papers provide a survey of the literature in NLP and SLP and the data to understand the trends and the evolution of research in this research community. This study can also be seen as a methodological framework for producing similar surveys for other scientific areas. The authors report on the major obstacles that appear during such processing. The first one are the errors that are due to the automatic processing of the full text of papers and in particular scanned content. The second obstacle is the lack of a consistent and uniform identification of authors, affiliations, conference titles, etc. which all require manual corrections by experts in the research area that is investigated.

## 3. Conclusion

The large number of studies on the use of scientific documents with bibliometric applications shows the growing interest of the bibliometric community in this subject. Since 2016, we have been maintaining the “Bibliometric-enhanced-IR Bibliography^1^” which is a bibliography of all scientific articles (workshops and journals) on this Research Topic. In 2018, two special issues closely related to this Research Topic were published. The first one is the special issue on “Bibliometric-enhanced information retrieval and natural language processing for digital libraries (BIRNDL)” in the *International Journal on Digital Libraries* (Mayr et al., 2018). The second one is “Bibliometric-enhanced Information retrieval and Scientometrics” in *Scientometrics* (Cabanac et al., 2018).

The articles published in this Research Topic contribute to the state of the art through theoretical discoveries, practical methods and technologies for the processing of scientific corpora involving full text processing, classification of citations but also their temporal representation, semantic analysis, text mining, and related topics. Taken together, these papers identify some of the new challenges in this area and pave the way for future theoretical frameworks.

The development of deep learning techniques is emerging in this field with approaches based on neural network models and can play a fundamental role in the exploitation of citations and their contexts in the scientific literature. While the development of neural network models requires large resources, the increasing number of datasets that are available today allows the implementation of this type of technology for the analysis of citations. Indeed, two of the articles in this Research Topic deal with the implementation of neural network models for citation analysis (Rodrigues Alves et al. and Nomoto), and other two with the construction and exploitation of a large scale corpus of papers (Mariani et al. and Mariani et al.).

## Author Contributions

All authors listed have made a substantial, direct and intellectual contribution to the work, and approved it for publication.

### Conflict of Interest Statement

The authors declare that the research was conducted in the absence of any commercial or financial relationships that could be construed as a potential conflict of interest.

## Footnotes

^1^https://github.com/PhilippMayr/Bibliometric-enhanced-IR_Bibliography/
